# The Neurocognitive and MRI Outcomes of West Nile Virus Infection: Preliminary Analysis Using an External Control Group

**DOI:** 10.3389/fneur.2018.00111

**Published:** 2018-03-27

**Authors:** Kristy O. Murray, Melissa S. Nolan, Shannon E. Ronca, Sushmita Datta, Koushik Govindarajan, Ponnada A. Narayana, Lucrecia Salazar, Steven P. Woods, Rodrigo Hasbun

**Affiliations:** ^1^Department of Pediatrics, Section of Pediatric Tropical Medicine, The National School of Tropical Medicine, Baylor College of Medicine, Texas Children’s Hospital, Houston, TX, United States; ^2^Department of Diagnostic and Interventional Imaging, The University of Texas Health Science Center at Houston, Houston, TX, United States; ^3^Department of Psychology, University of Houston, Houston, TX, United States; ^4^Department of Internal Medicine, The University of Texas Health Science Center at Houston, Houston, TX, United States

**Keywords:** West Nile virus, cortical thinning, regional brain atrophy, Repeatable Battery for the Assessment of Neuropsychological Status, neurocognitive outcomes, neurological outcomes

## Abstract

To understand the long-term neurological outcomes resultant of West Nile virus (WNV) infection, participants from a previously established, prospective WNV cohort were invited to take part in a comprehensive neurologic and neurocognitive examination. Those with an abnormal exam finding were invited for MRI to evaluate cortical thinning and regional brain atrophy following infection. Correlations of presenting clinical syndrome with neurologic and neurocognitive dysfunctions were evaluated, as well as correlations of neurocognitive outcomes with MRI results. From 2002 to 2012, a total of 262 participants with a history of WNV infection were enrolled as research participants in a longitudinal cohort study, and 117 completed comprehensive neurologic and neurocognitive evaluations. Abnormal neurological exam findings were identified in 49% (57/117) of participants, with most abnormalities being unilateral. The most common abnormalities included decreased strength (26%; 30/117), abnormal reflexes (14%; 16/117), and tremors (10%; 12/117). Weakness and decreased reflexes were consistent with lower motor neuron damage in a significant proportion of patients. We observed a 22% overall rate of impairment on the Repeatable Battery for the Assessment of Neuropsychological Status (RBANS), with impairments observed in immediate (31%) and delayed memory (25%). On MRI, participants showed significant cortical thinning as compared to age- and gender-matched controls in both hemispheres, with affected regions primarily occurring in the frontal and limbic cortices. Regional atrophy occurred in the cerebellum, brain stem, thalamus, putamen, and globus pallidus. This study provides valuable new information regarding the neurological outcomes following WNV infection, with MRI evidence of significant cortical thinning and regional atrophy; however, it is important to note that the results may include systemic bias due to the external control group. Considering no effective treatment measures are available, strategies to prevent infection are key.

## Introduction

With West Nile virus (WNV) now endemic throughout North America, with upwards of three million infected cases, it is a priority to document the long-term clinical outcomes (1–3). Among a large cohort of participants with a history of WNV infection in Houston, TX, USA, we found that 40% of those who presented with clinical disease continued to experience WNV-related morbidity up to 8 years postinfection (4). This percentage was up to 80% for those who initially presented with encephalitis. Sequelae varied in severity, with a higher than expected proportion developing chronic neurological and renal disease (5–10). Other shorter-term studies report neurocognitive impairment, debilitating fatigue, persistent neuromuscular paralysis, tremors, and depression as outcomes experienced by patients with a history of West Nile Neuroinvasive Disease (WNND) (7, 11–16). There are currently no published studies correlating neurocognitive evaluations to neuroimaging outcomes in patients with WNND.

MRI is the most sensitive neuroimaging technique for intracranial infections because of its superior soft tissue detail, sensitivity to subtle inflammatory processes, vascular imaging techniques, and functional imaging. Limited MRI data are available to evaluate the long-term effects of WNV, and published studies of brief case report describe changes during acute infection, with areas of interest including the basal ganglia, thalamus, pons, and lobar gray and white matter (17–19). The goal of this study was to understand the long-term neurological function impairment, neurocognitive deficits, and/or MRI abnormal findings in patients with a history of WNV infection.

## Materials and Methods

### Study Population

**Figure 1:**
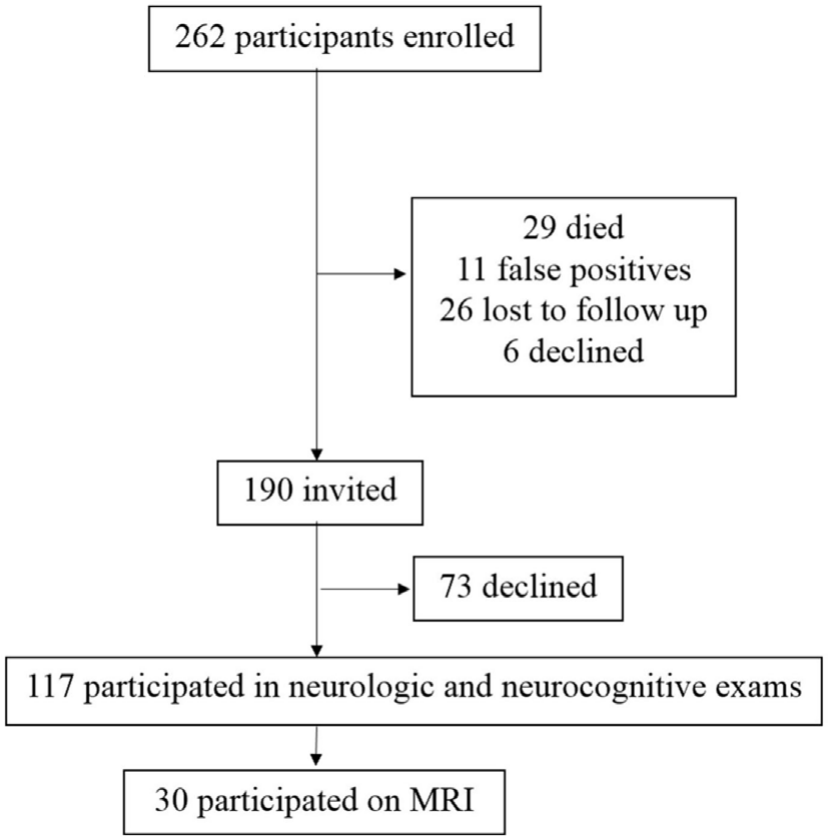
Study participant enrollment. From 2002 to 2012, a total of 262 patients with a history of West Nile virus (WNV) infection have been enrolled as research participants in this longitudinal cohort. Of the 262 potential participants, 190 were invited to participate. A total of 72 enrolled participants were not invited due to death (*n* = 29), additional tests determined a false positive WNV status (*n* = 11), lost-to-follow-up since initial enrollment (*n* = 26), or declined further participation since initial enrollment (*n* = 6). Of the 190 invited participants, 117 took part in the neurologic and the neurocognitive evaluations. Failure to take part was due to lack of desire to participate, inability to come to the Texas Medical Center for extensive testing, or unspecified reasons.

Following the first index WNV case in Houston, TX, USA in 2002, a cohort of WNV patients was established in collaboration with the City of Houston Health Department, Harris County Public Health, and the Gulf Coast Regional Blood Center. From 2002 to 2012, a total of 262 patients were enrolled as research participants in this longitudinal cohort. For the current study, 72 enrolled participants were unavailable due to death (*n* = 29), additional tests determined a false positive WNV status (*n* = 11), lost-to-follow-up since initial enrollment (*n* = 26), or self-withdrew from further participation since initial enrollment (*n* = 6). Of the 190 invited participants, 117 (62%) agreed to take part in the neurologic and the neurocognitive evaluations (Figure 1), and 30 took part in the MRI study (Figure 1). Failure to take part (*n* = 73) was due to inability to come to the Texas Medical Center for extensive testing, lack of desire to participate, or unspecified reasons.

### Standard Protocol Approvals, Registrations, and Patient Consents

This study was approved by Institutional Review Boards at both Baylor College of Medicine (H-30533) and the University of Texas Health Science Center at Houston (HSC-SPH-03-039). All participants provided informed written consent for their involvement in the study. Participants were registered as part of study protocol at the Memorial Hermann’s Clinical Innovation and Research Institute and University of Texas 3T MRI Center in Houston, TX, USA.

### Neurologic Examinations

To evaluate the sensory and motor aspects of infection, we used the collaborative antiviral study group neurological exam case report form utilized in the WNV immunoglobulin trial (20). Two physicians evaluated cranial nerves, deep tendon reflexes, fine touch sensory sensations, muscle strength, resting tremors, cerebellar dysmetria, and tone. Physical abilities were measured using the Barthel Index. Mental status was evaluated using the Glasgow Coma Scale and Glasgow Outcome Score (21, 22).

### Neurocognitive Examinations

To evaluate the psychological aspects of sequelae, neurocognitive evaluations were performed. These included multiple validated questionnaires and task-based exams to assess various aspects of cognitive functions. These evaluations were performed at Memorial Hermann’s Clinical Innovation and Research Institute, Houston, TX, USA. Quality of life and depression were measured using the Short Form-36 Health Survey and Becks Depression Index-II (BDI), respectively (23). The Weschler Abbreviated Scale of Intelligence (WASI) Matrix Reasoning subtest evaluated perceptual reasoning. Gesture to command tested apraxia (difficulty of motor planning to perform a task). The token test assessed aphasia (language impairment). The Galveston Orientation and Amnesia test (GOAT) measured attention and orientation (24). Finally, we administered the Repeatable Battery for the Assessment of Neuropsychological Status (RBANS), which provides index scores measuring immediate memory, visuospatial/constructional, language, attention and delayed memory (23).

### MRI

Participants with an abnormal neurological exam, neurological complaints, and/or and RBANS score less than 85 were invited to participate in the MRI studies. MRI studies were completed at the University of Texas at Houston MRI Facility using a 3T Philips Ingenia MRI scanner. The MRI protocol included multiple sequences. In this study, the analysis was based on MPRAGE 3D T1-weighted images. These images were acquired in the sagittal plane with an isotropic resolution of 1 mm. The other imaging parameters were: TR = 8.1 ms, TE = 3.7 ms, FOV = 256 mm × 256 mm, number of slices = 181. Thirty subjects consisting of 18 females and 12 males with an average age of 46 years (± 12) were compared to 30 scans on age- and gender-matched normal controls selected from the OASIS database^1^ for comparing regional atrophy and cortical thickness in the WNV subjects.

### Cortical Thickness Analysis

Cortical thickness analysis was carried out on the FreeSurfer^2^ software pipeline (version 5.1) (25, 26). The pipeline consists of a volumetric stream and a surface-based stream. Initially, all the volumes were registered to template space and then underwent intensity normalization. After that, a skull-stripping procedure based on a combination of a watershed algorithm and deformable template model was applied to extract the brain from the images. Following this, the output brain mask was labeled using a probabilistic atlas where each voxel in the normalized brain mask volume is assigned one of the following labels: cerebral white matter, cerebral cortex, lateral ventricle, inferior lateral ventricle, cerebellum white matter, cerebellum cortex, thalamus, caudate, putamen, pallidum, hippocampus, amygdala, accumbens area, third ventricle, fourth ventricle, brainstem, and cerebrospinal fluid (27).

The surface-based stream consists of a white matter segmentation followed by tessellation that identifies gray matter and white matter boundary and pial surfaces (25, 27, 28). Likely, white matter points were identified using voxel intensity and neighborhood intensity information. White and pial surfaces were constructed after refining from initial surfaces generated for each hemisphere. The thickness is defined as the average of the distance between the surface and the gray and white matter (GM-WM) boundary and the distance between the GM-WM boundary and the surface (28).

FreeSurfer implements a technique for automatically assigning a neuroanatomical label to each location on a cortical surface. It uses both geometric information derived from the cortical model and neuroanatomical convention to form a complete labeling of the cortical gyri and sulci. FreeSurfer uses the Desikan-Killiany Atlas and the Destrieux Atlas. For this study, the labels based on Desikan-Killiany atlas were used for cortical thickness measurements, and maps were generated for both the participant and control datasets.

### Regional Atrophy Analysis

3D T1-weighted images underwent preprocessing that included skull-stripping and intensity non-uniformities correction. Non-linear symmetric diffeomorphic registration technique (29, 30) was applied to co-align all the pre-processed 3D T1 images to International Consortium for Brain Mapping (ICBM) template.^3^ For each subject, a 3D diffeomorphic map was generated during non-linear registration which was used to calculate Jacobian determinant at each voxel (31).

### Statistical Analyses

Descriptive statistics were performed to describe the outcome variables of the cross-sectional study on prospectively followed participants. Univariate analyses were then conducted with the Pearson’s X2 test or Fisher’s exact test to identify factors of neurological and neurocognitive exams that were significant between clinical descriptions of WNV cases. For cortical thickness, FreeSurfer’s inbuilt generalized linear model (GLM) was used to determine group differences between WNV cases and controls. Using the GLM, uncorrected group difference maps for cortical thickness were generated. These maps represent voxel-wise differences in cortical thickness between the WNV cases and selected controls. Following this, a multiple comparison Monte-Carlo simulation was performed with a voxel-wise threshold of *p* = 0.05 and a cluster-wise threshold of *p* = 0.05, using 5,000 iterations. The resulting output maps represent the significant regions of differences between the two groups.

For regional atrophy studies, atrophy of any region on 3D T1 in comparison to that of ICBM template were determined by the positive and negative values of average JDs of the region. Logarithmic of JDs at each voxels within intracranial brain were calculated following the normalization of JDs that accounts for the brain size. These values at each voxel were obtained for both participants and healthy controls. Healthy control data were obtained as described above. Group analysis of the average normalized logarithmic JDs between participants and controls for each voxel was done using statistical tools available in Statistical Parametric Mapping. The voxels with significant differences were obtained based on the false discovery rate of 0.05 and were considered as the voxels representing atrophy.

We were also interested in analyzing the association between neurocognitive deficits and cortical thickness. To reduce Type I error, we examined pairwise correlations between the RBANS total score and the cortical thickness regions of interest using Pearson product moment coefficients. Given the exploratory nature of these analyses, we set the critical alpha level at 0.01.

## Results

Table 1 shows the demographics of our study population, which consisted of mostly white, non-Hispanic (83%) individuals, and an almost equal proportion of males (53%) and females. Mean age at the time of exam was 57 years (range 18–89 years). Most participants had graduated high school (98%), and almost half (47%) were employed full time at the time of their neurological exam. Almost one-third of participants were hypertensive (32%), and 15% were diabetic. The majority of participants (77%) were four or more years past their acute WNV infection. Participants evaluated had a history of asymptomatic disease (23%), West Nile Fever (WNF) (33%), or WNND (44%).

**Table 1:** Baseline demographics of 117 adults with history of West Nile virus infection who underwent a complete neurological examination.^a^

Baseline variables	*N* = 117 (%)
Mean age at exam (range)	57 years (18–89)
Male gender	63 (53)
Race/ethnicity	
White, non-Hispanic	98 (83)
White, Hispanic	10 (8)
Black	6 (6)
Asian	3 (3)
Acute clinical presentation at time of West Nile virus (WNV) infection			
Encephalitis	35 (30)
Meningitis	16 (14)
Uncomplicated fever	39 (33)
Asymptomatic	27 (23)
Education level	
High school degree	42 (36)
Undergraduate degree	40 (34)
Graduate degree	33 (28)
Less than high school degree	3 (2)
Employment status at time of exam	
Full-time employment	55 (47)
Retired	38 (32)
Unemployed, disability, or other employment	22 (19)
Part-time employment	3 (2)
Comorbid conditions	
Hypertension	38 (32)
Diabetes	17 (15)
Time between WNV infection and neurologic exam	
0–3 years	26 (22)
4–7 years	46 (39)
8–11 years	45 (38)

*^a^Neurologic exam included evaluation of cranial nerves, deep tendon reflexes, fine touch sensory sensations, muscle strength, resting tremors, cerebellar dysmetria, and tone*.

Across the entire cohort, almost half (49%; 57/117) of participants had some type of abnormal neurological exam finding, with most abnormalities being unilateral in nature (Tables 2 and 3). The most common abnormalities included abnormal (decreased) strength (26%; 30/117), abnormal reflexes (14%; 16/117), and tremors (10%; 12/117). Decreased strength was more common among core muscle groups than in the extremities, with the exception of finger strength, and was associated with decreased reflexes in 16 out of 30 patients consistent with lower motor neuron involvement. Abnormal reflexes were consistent among all five muscle groups. Finally, tremors were more common among the upper body than the lower body, which was distinct from other findings. There was no statistical difference between time from infection (5 years or more vs. less than 5 years) and having an abnormal neurological examination (*p* = 0.73).

**Table 2:** Neurological exam findings in 117 adults with West Nile virus infection by acute clinical picture.^a^

		Non-neuroinvasive		Neuroinvasive^a^			
Area of examination	All participants *n* = 117	No symptoms *n* = 27	West Nile fever *n* = 39	Meningitis *n* = 16	Encephalitis *n* = 35	Odds ratio^b^ (95% confidence interval)	*p*-Value
Abnormal neurological	57	7	20	11	19	1.8 (0.8–3.8)	NS^c^
Abnormal cranial nerve	8	4	4	0	0	Undefined	NS
Abnormal reflexes^d^	16	0	4	3	9	4.2 (1.2–14.6)	0.02
Abnormal sensory	8	0	3	4	1	1.9 (0.4–8.8)	NS
Abnormal strength	30	1	10	4	12	2.5 (1.1–6.3)	0.04
Tremors	12	2	3	1	6	1.5 (0.4–5.4)	NS
Abnormal cerebellar^e^	2	0	1	0	1	0.7 (0.03–13.9)	NS
Abnormal tone	1	0	1	0	0	Undefined	NS

*^a^Meningitis, encephalitis, or acute flaccid paralysis presentation*.

*^b^Adjusted for age, gender, and history of diabetes mellitus*.

*^c^Non-significant p-value of >0.05*.

*^d^All reflexes were decreased*.

*^e^Cerebellar ataxia*.

**Table 3:** Neurological exam findings on 117 adults with West Nile virus infection.

Area of examination	Total number with abnormal findings (%)	Number with abnormal findings on one side of the body (%)	Number with abnormal findings on both sides of the body (%)
Cranial nerves			
Ptosis	3/117 (3)	0/117	3/117
Visual field deficit	5/116 (4)	1/116	4/116
Extraocular movements	0/117 (0)	0/117	0/117
Facial palsy	0/117 (0)	0/117	0/117
Abnormal reflexes (decreased)			
Biceps	10/117 (9)	1/117	9/117
Brachioradialis	10/117 (9)	1/117	9/117
Triceps	12/117 (10)	0/117	12/117
Quadriceps	9/116 (8)	3/116	6/116
Gastrocnemius	5/116 (4)	1/116	4/116
Abnormal sensation			
Upper extremities	3/117 (3)	2/117	1/117
Lower extremities	5/115 (4)	1/115	4/115
Face	0/117 (0)	0/117	0/117
Decreased strength			
Deltoid	7/117 (6)	1/117	6/117
Biceps	6/117 (5)	1/117	5/117
Brachioradialis	5/117 (4)	0/117	5/117
Triceps	8/117 (7)	3/117	5/117
Iliopsoas	6/115 (5)	1/115	5/115
Quadriceps	7/116 (6)	2/116	5/116
Hamstrings	5/116 (4)	0/116	5/116
Grip	7/117 (6)	3/117	4/117
Foot dorsiflexion	5/115 (4)	2/115	3/115
Foot plantar flexion	2/114 (2)	0/114	2/114
Toe wiggling	1/114 (0.9)	0/114	1/114
Arm drifts	3/117 (3)	2/117	1/117
Firmly hold paper between third and fourth fingers	17/117 (15)	5/117	12/117
Tremors			
Face	2/117 (2)	1/117	1/117
Upper extremity	11/117 (9)	4/117	7/117
Lower extremity	0/117 (0)	0/117	0/117
Abnormal cerebellar exam			
Finger to nose	2/117 (2)	0/117	2/117
Heel to shin	1/116 (0.9)	0/116	1/116

The frequencies of gross neurocognitive and ADL abnormalities were quite low, with fewer than 5% of the cohort producing abnormal scores on the GCS, GOS, GOAT, and Barthel Index (Tables 4 and 5). Similarly, fewer than 5% of the sample had IQ scores in the Borderline or extremely low range (i.e., <80). However, the frequency of milder higher-order neurocognitive and mood disturbances was somewhat higher. Using a 1SD cutpoint derived from published normative standards, we observed a 22% overall rate of impairment on the RBANS, with the domains of immediate (31%) and delayed memory (25%) being the most frequently impaired. Seven percent of participants had RBANS total scores in the moderately to severely impaired range (<2SD). There was no significant difference between timing of the exam (five or more years since infection versus less than 5 years) and low RBANS score (*p* = 0.16). Consistent with previous findings, approximately one-fifth of the sample reported current depressed mood using a conservative BDI cutoff score of 17, which is commonly recommended in medical and neurological populations (32).

**Table 4:** Neuropsychological exam findings in adults with West Nile virus infection by acute clinical presentation.

Domain	Non-neuro *n* = 76 (%)	Neuroinvasive *n* = 35 (%)	Univariate *p*-value	Multivariate^a^ *p*-values
**Neurological Screening**														
Glasgow Coma Scale	15.0 (0.0)	15.0 (0.0)	NS	–
Glasgow Outcome Scale	5.0 (0.0)	4.9 (0.0)	NS	–
GOAT	96.9 (0.4)	94.8 (0.6)	0.004	0.030
**Neurocognitive**				
WASI matrix reasoning	52.5 (1.2)	50.7 (1.8)	NS	–
RBANS total	100.2 (2.0)	91.0 (3.1)	0.013	0.060
Immediate memory	94.3 (2.0)	86.8 (3.2)	0.050	NS
Delayed memory	97.6 (2.0)	91.1 (3.1)	NS	–
Visuospatial	108.0 (1.8)	98.2 (2.8)	0.004	0.011
Language	96.9 (1.2)	93.5 (1.9)	NS	–
Attention	104.1 (2.3)	99.9 (3.5)	NS	–
Token test	33.9 (0.4)	33.6 (0.6)	NS	–
**Mood**				
Beck depression inventory	8.9 (1.2)	10.0 (1.8)	NS	–
**Functioning**				
Barthel ADL Index	100.0 (0.6)	95.9 (0.9)	<0.001	0.004
Short Form-36	99.4 (1.2)	96.3 (1.8)	NS	–

*GOAT, Galveston Orientation and Amnesia Test; RBANS, Repeatable Battery for the Assessment of Neuropsychological Status; WASI, Wechsler Abbreviated Scale of Intelligence*.

*^a^Analyses controlled for age and education*.

**Table 5:** Neuropsychological exam findings in adults with West Nile virus infection.

Domain	Mean (SD)	Range	Clinically abnormal (%)
Neurological screening			
Glasgow Coma Scale (*n* = 117)	15.0 (0.0)	15–15	0.0^a^
Glasgow Outcome Scale (*n* = 117)	5.0 (0.2)	4–5	3.4^b^
GOAT (*n* = 116)	96.2 (3.6)	75–100	0.8^c^
Neurocognitive			
WASI matrix reasoning (*n* = 110)	51.3 (10.9)	20–76	3.6^d^
RBANS total (*n* = 112)	97.0 (17.7)	54–148	22.3^e^
Immediate memory	91.6 (18.2)	40–136	31.3^e^
Delayed memory	95.3 (17.9)	44–127	25.0^e^
Visuospatial/constructional	104.5 (16.5)	60–131	13.4^e^
Language	95.3 (11.0)	57–120	9.8^e^
Attention	102.9 (19.4)	53–150	17.9^e^
Token test (*n* = 112)	33.6 (3.3)	21–36	–
Mood			
Beck Depression Inventory (*n* = 116)	9.4 (10.3)	0–43	22.4^f^
Functioning and quality of life			
Barthel ADL Index (*n* = 117)	98.7 (5.3)	60–100	2.6^g^
Short form-36 (*n* = 115)	98.4 (10.5)	50–126	–

*GOAT, Galveston Orientation and Amnesia Test; RBANS, Repeatable Battery for the Assessment of Neuropsychological Status; WASI, Wechsler Abbreviated Scale of Intelligence*.

*^a^GCS < 13*.

*^b^GOS < 5*.

*^c^GOAT < 76*.

*^d^WASI T scores < 30*.

*^e^RBANS standard scores < 84*.

*^f^BDI raw scores ≥ 17*.

*^g^Barthel scores < 90*.

**Figure 2:**
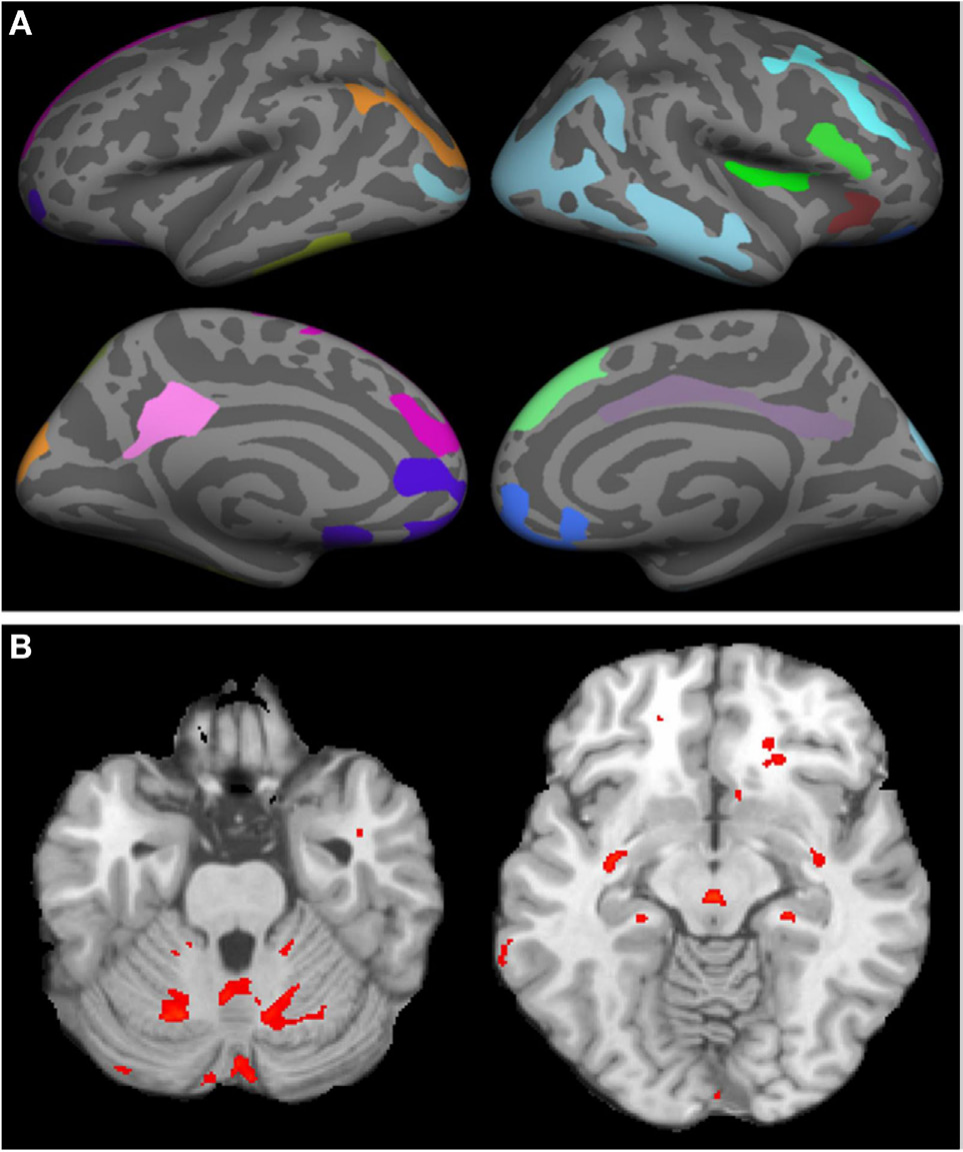
Cortical thinning and regional atrophy analyses. **(A)** Volumetric MRI. Left (first column) and right (second column) hemispheres showing cortical thinning in West Nile infected individuals as compared to a database of MRIs from healthy controls with lateral (top row) and medial (bottom row) views. Colored regions indicate areas of thinning. In the left hemisphere, parts of the posterior cingulate cortex (pink), parts of the superior frontal cortex and medial-orbito frontal region (magenta), anterior cingulate cortex and inferior frontal cortex (violet blue), parts of the cuneus (orange) and para hippocampal region (pale green) were identified. In the right hemisphere, parts of the middle and inferior temporal cortex, and supramarginal region (light blue), inferior frontal region and insular cortex (bright green and red), parts of the superior frontal cortex (light green, blue, and violet), cingulate cortex (light purple), and inferior frontal region (blue) were identified. **(B)** Tensor-based morphometry (TBM) analysis of regional atrophy. Regions with regional atrophy as detected by TBM are identified in red.

**Figure 3:**
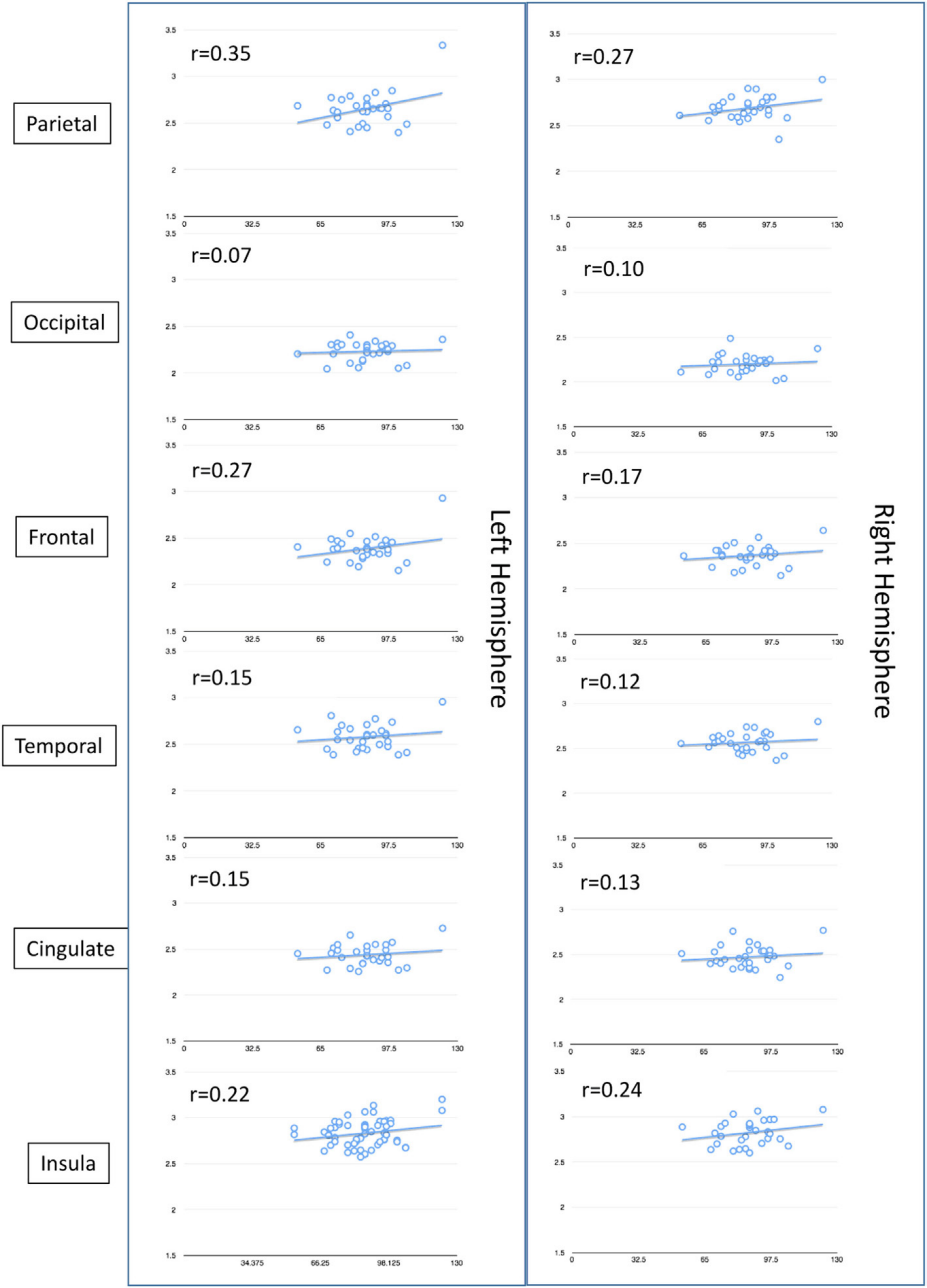
Non-pairwise scatterplots of raw data compairing total Repeatable Battery for the Assessment of Neuropsychological Status score (*y*-axis) to cortical thickness (*x*-axis) by region and hemisphere of the brain among the 30 West Nile virus participants who underwent MRI.

On MRI, WNV participants showed significant cortical thinning as compared to age- and gender-matched controls (Figure 2A; raw data in Tables S1 and S2 in Supplementary Material). Significant cortical thinning in the left hemisphere (LH) included parts of the posterior cingulate cortex (pink), parts of the superior frontal cortex and medial-orbito frontal region (magenta), anterior cingulate cortex and inferior frontal cortex (violet blue), parts of the cuneus (orange) and para-hippocampal region (pale green), and in the right hemisphere (RH) included parts of the middle and inferior temporal cortex, and supramarginal region (light blue), inferior frontal region and insular cortex (bright green and red), parts of the superior frontal cortex (light green, blue, and violet), cingulate cortex (light purple), and inferior frontal region (blue). Lower neurocognitive functioning as determined by total RBANS score was associated with cortical thinning in regions of the LH only: caudal middle frontal gyrus (*r* = 0.49, *p* = 0.008), rostral middle frontal gyrus (*r* = 0.49, *p* = 0.007), and supramarginal gyrus (*r* = 0.51, *p* = 0.004). Scatterplots comparing cortical thickness and total RBAN score by region and hemisphere among WNV participants can be found in Figure 3.

Tensor-based morphometry analysis of participants’ scans vs. control scans revealed significant regional atrophy in the overall cohort of 30 WNV participants that underwent MRI (Figure 2B). Significant regional atrophy was identified in the cerebellum, brain stem, thalamus, putamen, and globus pallidus. No significant correlation between a low RBANS score and these regions was identified.

## Discussion

This study contributes one of the largest cross-sectional examinations of the neurological and neurocognitive outcomes post-WNV infection, and the only known study to link these quantitative measures to MRI findings, allowing for valuable insight into the pathology of infection. These findings can be useful in the clinical management of patients with a history of WNV, particularly those with WNND.

Results of the neurological exam showed weakness and decreased reflexes consistent with lower motor neuron damage in a substantial proportion of patients (Tables 2 and 3) and identified WNND participants to be at an increased risk for these outcomes, findings consistent with current literature. Weatherhead et al. described abnormal reflexes and muscle weakness as common abnormalities in a prospective study that evaluated WNND and WNF participants in the Houston WNV cohort 1–3 and 8–11 years after their initial WNV infection (10), with results indicating that participants with a history of WNND with encephalitis were more likely to have persistent and progressive abnormalities.

When comparing the results of the neurocognitive exams between neuroinvasive and non-neuroinvasive cases, we see a significant difference in only a few of the measures (GOAT, RBANS total score, RBANS immediate memory, RBANS visuospatial, and Barthel ADL Index) (Table 4). A larger than expected number of the WNF cases (20%) had observable deficits. In order to meet the case definition of WNND, it was required to have a lumbar puncture procedure with evidence of pleocytosis; therefore, it is possible that these cases were misclassified as WNF, when indeed, they had WNND. Newer guidelines now permit the diagnosis of encephalitis without lumbar puncture if patients present with altered mental status for greater than 24 h with no alternative explanation and meet at least three of the following criteria: fever 38 C or above, generalized or partial seizures unrelated to a preexisting seizure disorder, new onset of focal neurologic findings, abnormality of brain parenchyma on neuroimaging, abnormality in electroencephalography (33). Since we do not have this information for all participants, we cannot reevaluate their classification in this study. We acknowledge this limitation and that the significance of WNND as a risk factor for sequelae may be underrepresented in this study population. Also, none of our patients evaluated had WNV-associated acute flaccid paralysis, which is known to have poorer clinical outcomes than other presentations of WNND (5, 6). It would be valuable in future studies to further distinguish neurological and neurocognitive outcomes among the different clinical presentations of WNND (meningitis vs. encephalitis vs. AFP), since these different presentations have varying severity of morbidity (4).

MRIs to evaluate cortical thinning and atrophy were performed only on participants with ongoing neurological complaints and/or a low RBANS score. When compared to age- and gender-matched controls from the OASIS database, cortical thinning was identified in the frontal and limbic lobes of both the left and RHs, with small regions of the temporal and parietal lobes also indicated in the RH. Interestingly, participants with a low RBANS score also had significant thinning within regions of the LH. Significant regions were within the middle frontal gyrus and supramarginal gyrus, both within the parietal lobe. The parietal lobe is responsible for sensation and perception and integrating sensory input, areas that were identified to be weakened in some participants. The lack of overlap between regions affected by WNV compared to controls and cortical thinning correlates of the RBANS analysis of WNV participants may be a simple artifact of measurement. Namely, the RBANS is a brief neurocognitive screening measure and does not include tasks that assay the frontal regions identified in the cortical thinning analysis. This lack of overlap warrants additional neurocognitive measures in future studies with larger participant groups.

Although most WNV-infected participants were recruited in the Houston area, we were not able to match controls from OASIS based on location for this study. Additionally, the OASIS database contains data from controls scanned with a different machine with regards to both field strength and manufacturer when compared to our scans (1.5T Siemens and 3T Philips, respectively). While these differences could impact our findings and lead to systematic bias, a recent publication from our group (34) found that when examining the effect of field strength between 1.5 and 3T, the cortical thickness showed field dependence only in certain regions that include posterior cingulate and parahippocampal regions in the LH and middle temporal cortex in the RH. In this current study on WNV, we observed significant cortical thinning in the LH that included parts of the posterior cingulate cortex (pink), parts of the superior frontal cortex and medial-orbito frontal region, anterior cingulate cortex and inferior frontal cortex, parts of the cuneus and para-hippocampal region, and in the RH included parts of the middle and inferior temporal cortex, and supramarginal region, inferior frontal region and insular cortex, parts of the superior frontal cortex (light green, blue, and violet), cingulate cortex, and inferior frontal region. Thus, many regions in which cortical thickness differences were observed in WNV patients did not show field dependence. Nonetheless, predicting systematic bias when using different scanners is difficult and this is an important limitation of our study. It will be crucial to confirm these results in future studies by concurrently enrolling and imaging control participants using the same scanners.

Regional atrophy was noted in the cerebellum, brain stem, thalamus, putamen, and globus pallidus of WNND patients who were not apparent in controls. These regions clearly mediate functions that are altered in WNND patients. Cerebellitis has only been described in one case report of a 10-year-old child (35). Infectious cerebellitis is most commonly caused by other etiologies such as JC virus, varicella zoster virus, and *Listeria monocytogenes* (36). The most common causes of brain stem/rhomboencephalitis are *L. monocytogenes* and Enterovirus 71 (37). WNV can occasionally have brain stem involvement (38). Encephalitis with thalamic and basal ganglia involvement should raise the possibility of respiratory viruses and WNV infection (39, 40). This thalamic and basal ganglia involvement is responsible for the facial and upper extremity tremors, motor weakness, and abnormal reflexes seen in WNND (41). Although regional atrophy did not statistically correlate with the RBANS total score, atrophy in WNV participants compared to controls still provides pathological insights into the impact of WNND disease. Larger sample sizes within WNND with encephalitis, meningitis, and/or acute flaccid paralysis would allow for further analysis of the effects of WNND and its specific clinical syndromes on regional atrophy. Also, serial MRI scans from acute disease onset to later timepoints would allow for documentation of atrophy and cortical thinning over time. Unfortunately, we were limited in this study to make comparisons to prior MRIs, as we only had MRI result data on six patients from the acute phase of illness, all of whom had a “normal” result provided.

Understanding the long-term complications of WNV infection are important to improving clinical outcomes and decreasing the costs on our health systems. A 2014 study determined that it can cost up to $400,000 per patient to treat WNV sequelae, not including the cost of care during the acute phase of illness (42). This study provides new insights of long-term sequelae by comparing neurological, neuropsychological, and MRI results of affected patients 3–8 years after initial infection. Although some correlations of functional evaluations and MRI were not statistically significant, the biological relevance of the alterations identified *via* MRI are interesting. For the first time, this study identified brain areas of interest, such as the cerebellum, putamen, thalamus, supramarginal gyrus, and middle frontal gyrus, in patients continuing to experience neurological complications. These data will be crucial for future studies regarding the progression of WNV disease and associated long-term complications. Afflicted regions also suggest the damage pattern could be similar to what is seen in traumatic brain injury. At this time, with no specific preventive or therapeutic options to treat WNV infection other than supportive care, continuing these types of large-scale and long-term prospective studies will allow us to generalize expected consequences of infection. In turn, this allows for proactive treatment of sequelae that would otherwise lower a patients’ quality of life. Ultimately, public health efforts to prevent infection are critical, as many of the issues identified in study participants are not reversible.

## Ethics Statement

This study was approved by Institutional Review Boards at both Baylor College of Medicine (H-30533) and the University of Texas Health Science Center at Houston (HSC-SPH-03-039). All participants provided informed written consent for their involvement in the study. Participants were registered as part of study protocol at the Memorial Hermann’s Clinical Innovation and Research Institute and University of Texas 3T MRI Center in Houston, TX, USA.

## Author Contributions

Conception and design of study: KM, PN, SW, and RH. Acquisition and analysis of data: KM, MN, SR, SD, KG, LS, SW, and RH. Manuscript/figure drafting: KM, MN, SR, KG, PN, SW, and RH.

## Conflict of Interest Statement

The authors declare that the research was conducted in the absence of any commercial or financial relationships that could be construed as a potential conflict of interest. The reviewer BS and handling Editor declared their shared affiliation.

## Footnotes

^1^https://www.oasis-brains.org/.

^2^http://surfer.nmr.mgh.harvard.edu/fswiki.

^3^http://www.loni.usc.edu/ICBM/Downloads/Downloads_ICBMtemplate.shtml.
